# Supplementary material for: A Biologically Interfaced Evolvable Organic Pattern Classifier
Source: Adv Sci (Weinh). 2023 Mar 19;10(14):2207023. doi: 10.1002/advs.202207023 (PMC10190637; doi:10.1002/advs.202207023)
Supplement: Supplementary file 1 — Supporting Information [file ADVS-10-2207023-s001.pdf]

Supporting Information

A biologically interfaced evolvable organic pattern classifier

Jennifer Y. Gerasimov, Deyu Tu, Vivek Hitaishi, Padinhare Cholakkal Harikesh, Chi-Yuan Yang, Tobias Abrahamsson, Meysam Rad, Mary J. Donahue, Malin Silverå Ejneby, Magnus Berggren, Robert Forchheimer*, Simone Fabiano**

J. Y. Gerasimov, D. Tu, V. Hitaishi, P. C. Harikesh, C.-Y. Yang, T. Abrahamsson, M. Rad, M. J. Donahue, M. Berggren, S. Fabiano
Laboratory of Organic Electronics, Department of Science and Technology, Linköping University, Norrköping SE-60174, Sweden. E-mail: jennifer.gerasimov@liu.se, simone.fabiano@liu.se

M. Silverå Ejneby
Department of Biomedical Engineering, Linköping University, Linköping SE-581 83, Sweden

R. Forchheimer
Department of Electrical Engineering, Linköping University, Linköping SE-581 83, Sweden.
E-mail: robert.forchheimer@liu.se

Keywords: organic electrochemical transistors, neuromorphic, synaptic transistors, organic electronics, conducting polymers, electropolymerization, evolvable electronics

The Least Mean Squares Algorithm

The purpose of the LMS algorithm is to minimize the error between the class labels (often chosen as +1 and -1) and the weighted sums ("regression values") generated by the network. The expectation is that this procedure should also minimize the classification error rate when the threshold circuit is applied. This is not always the case as the two criteria are different. However, the LMS optimization will converge also for patterns which are not linearly separable. This is in contrast to the Perceptron learning algorithm which is based on minimizing the error rate.[1]

The error that is considered in the LMS algorithm is the sum of the squared differences between the regression values $r_j = \sum_i w_i V_{ij}$ and the class labels:

$$e^2 = \sum_j \left(r_j - l_j\right)^2 \qquad \text{(S1)}$$

where w_i is the i:th weight, V_{ij} is the i:th component of a labelled input pattern V_j (being part of the "training set") and l_j is its label. As is seen the error is a quadratic function. Thus, the weights that minimize the error can be solved for analytically. This is a tedious procedure however that requires solving a linear equation system with as many equations as there are weights. It also requires that all the training samples are available simultaneously.

In contrast, the LMS algorithm iterates over the training patterns to approach the correct solution. Not only is the computational burden distributed over time, but the procedure allows that new patterns and/or changed labelling can be introduced to facilitate adaptivity. The basic idea is the following.

For each training pattern the weight vector W is adjusted to bring the regression value r_j closer to the label associated with that pattern. This can be expressed as:

$$W[j+1] = W[j] + step * \left(l_j - r_j\right) * V_j \qquad \text{(S2)}$$

where step controls the convergence rate. As an example, consider a case where the label is +1 for a certain input pattern while the regression value is less than 1. Then, all weights which are multiplied by a positive input should be increased while those which are multiplied by a negative input should be decreased. Altogether, this will make the new regression value come closer to +1. A similar procedure is done for an input pattern which is labelled -1. But here, the adjustment of the weights will be reversed. The amount which is added to each weight should be small enough not to cause overshoots but rather a smooth convergence. Following Widrow's ADALINE implementation [10a] we will only consider binary input patterns. Each pattern component is assumed to have the value 1 or -1.

Read voltage scaling

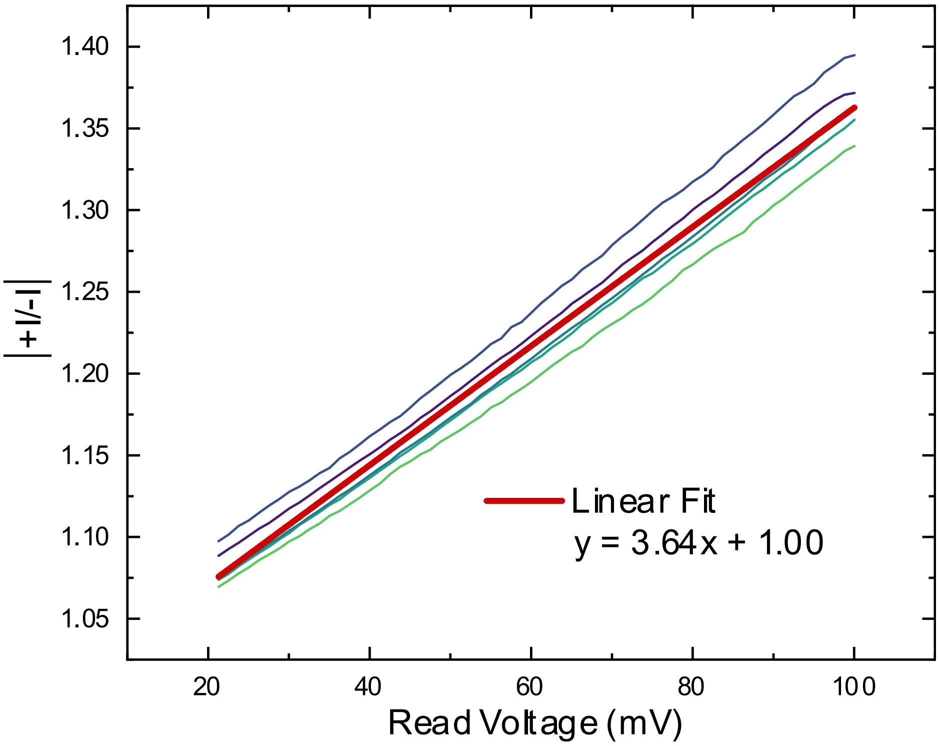

Figure S1. Current ratio between positive and negative voltages for a set of devices of varying conductance. Data taken from Figure 2e.

A non-destructive read can be obtained by keeping potential differences between the source/drain terminals and the gate well below 0.2 V. However, in read mode, it is very important that the positive and negative components of a given pixel are modulated so that only the difference in the effective channel volume rather than any difference in the redox state of the ETE-S channel is measured by the amp-meter. This is obtained by introducing an offset coefficient by which we multiply the negative input voltage to compensate for the reduction in the doping level. The offset coefficient is calculated from the IV data in Figure 2e by normalizing the positive drain current by the negative drain current for all drain voltages and extracting a linear fit that best accommodates all conductance states. From the fit, we can find $-V = +V\left|\frac{+I}{-I}\right|$ to calculate the appropriate negative read voltage $-V$.

Expanded Figure 2c

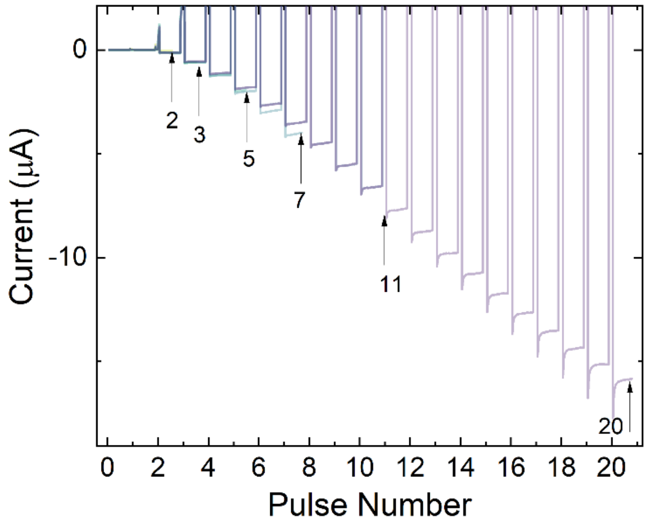

Figure S2. Inset of Figure 2c highlighting the current increase during the refractory period.

State Retention

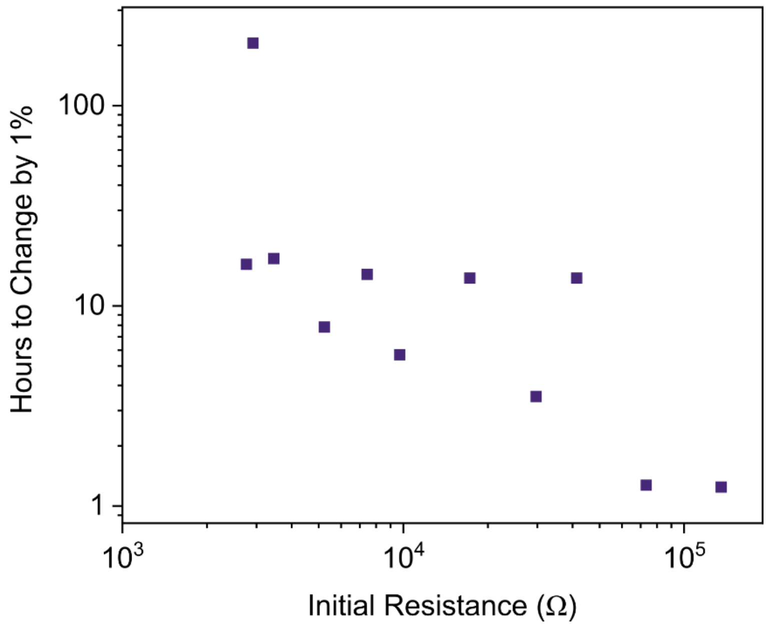

Figure S3. Time required for the current to change by 1%, as a function of the channel resistance. The highest point (206 hours) was excluded as an outlier to obtain an average time of 9.5 ± 6.3 hours.

Touchpad setup

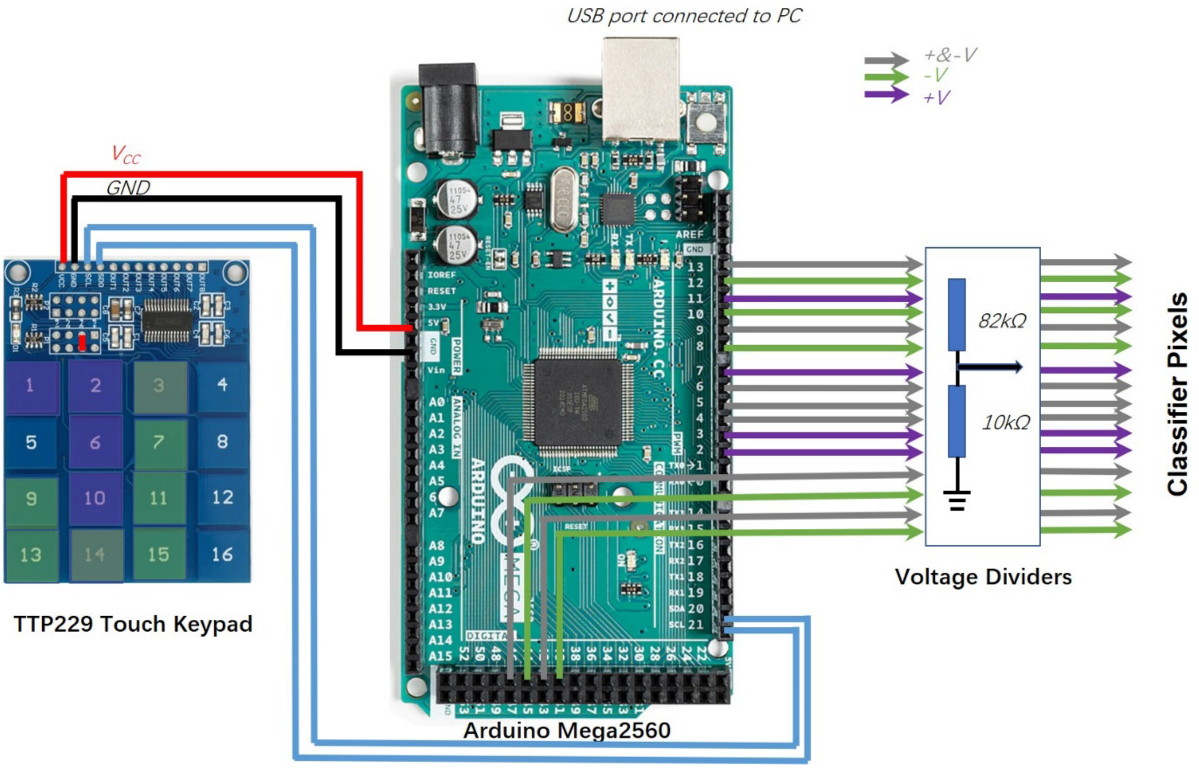

Figure S4. The touchpad setup for the "T/J" classifier training. The Arduino Mega 2560 microcontroller board is used to sample the touching signals from the keypad and deliver training pulses to the classifier through the voltage dividers.

OECN characterization

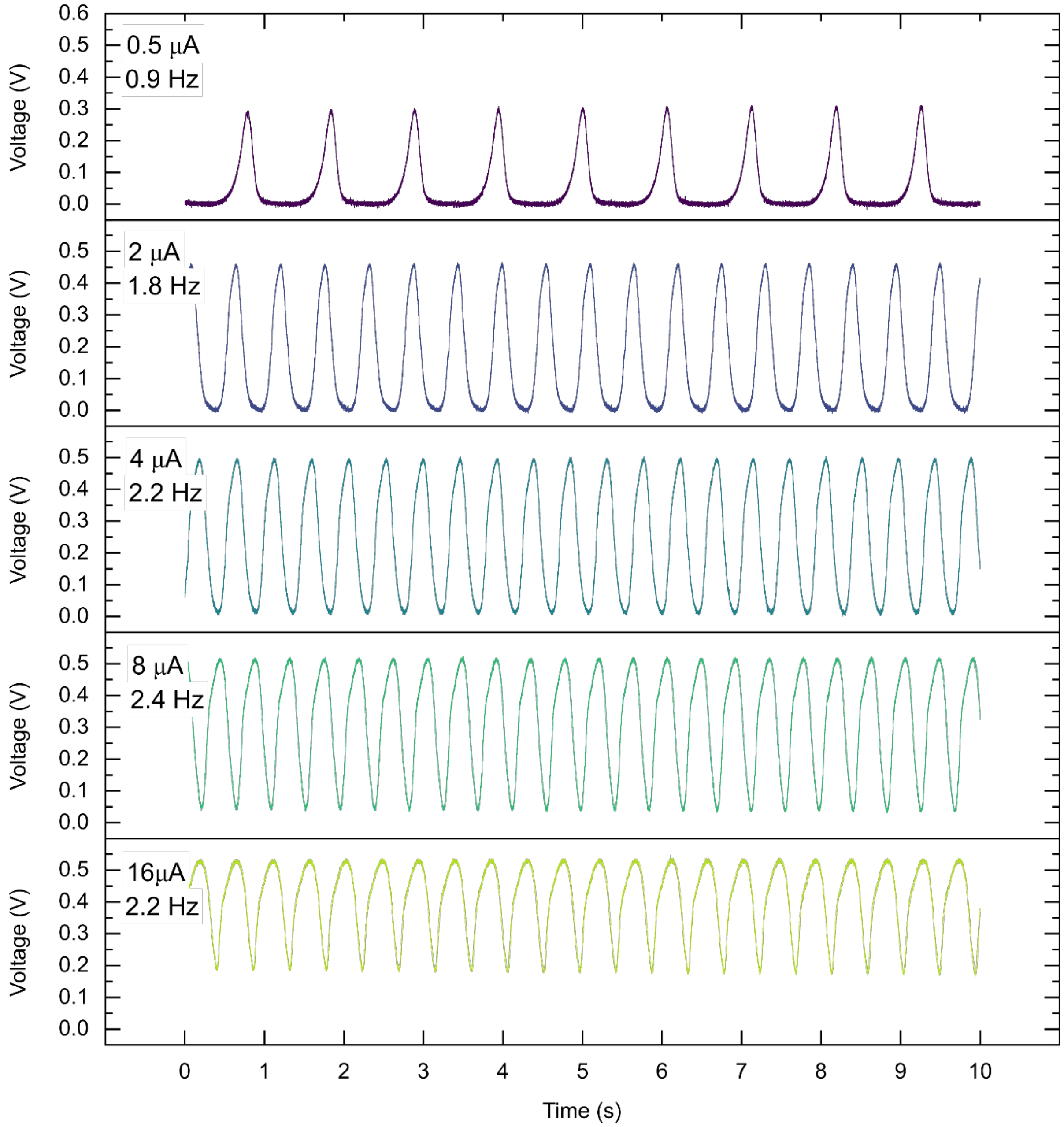

Figure S5. Neuron spiking behavior at a range of input currents.

Classifier Measurement Setup

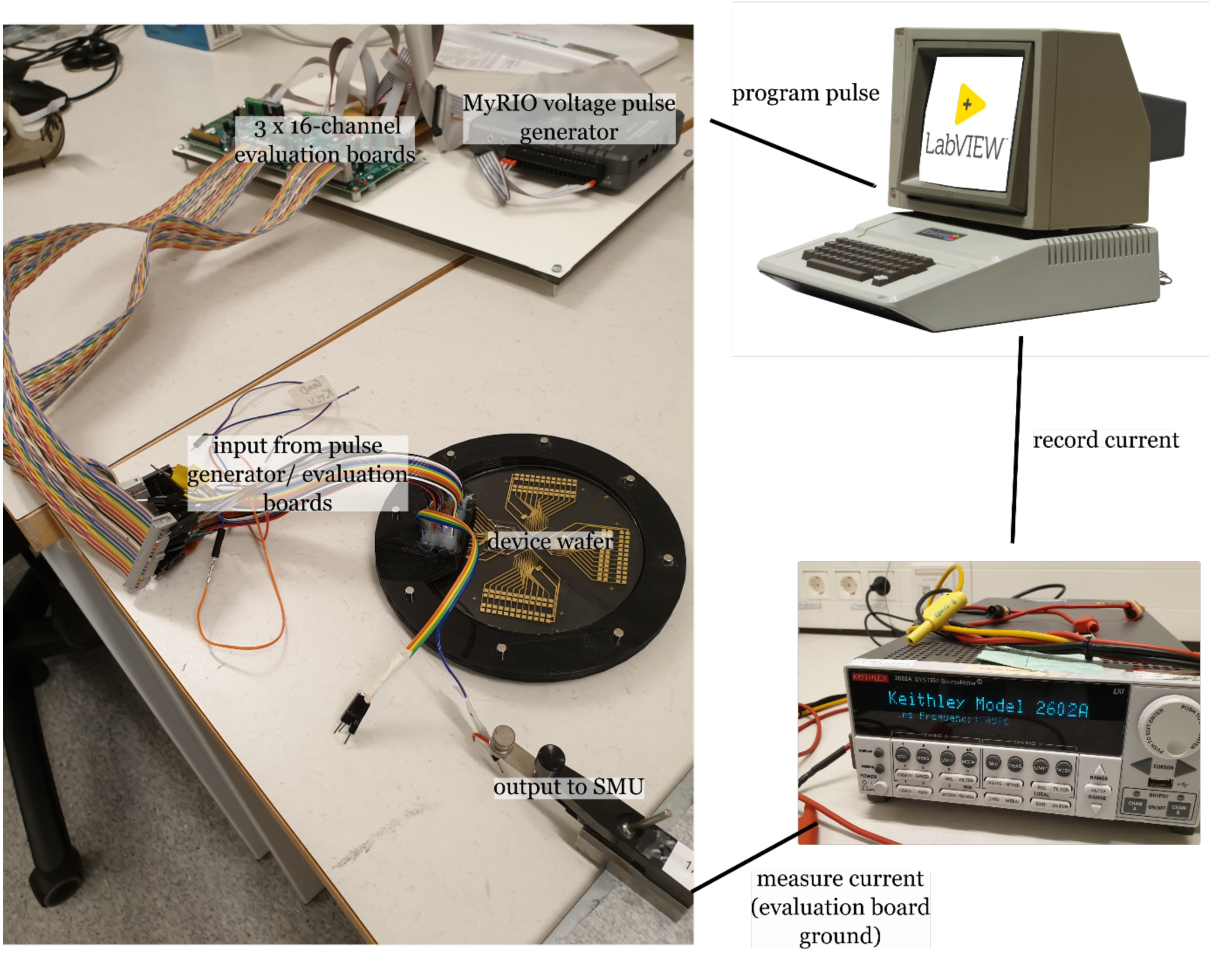

Figure S6. Classifier measurement setup.

Transimpedance Amplifier

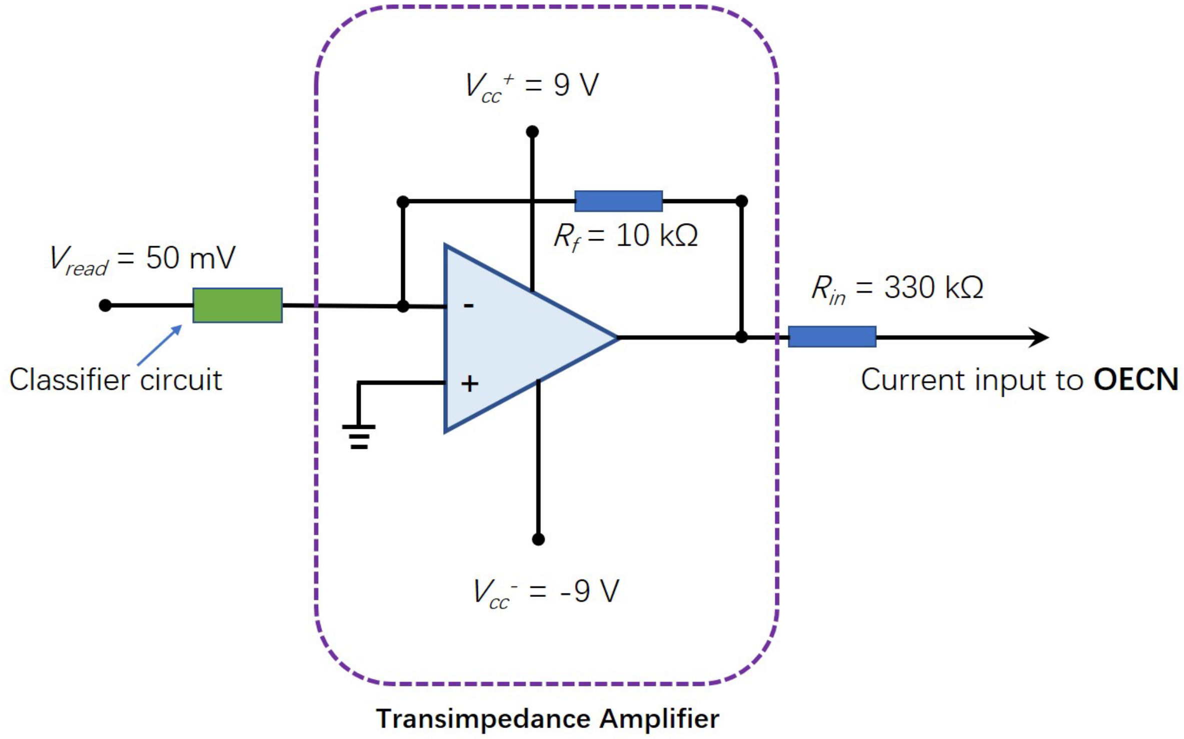

Figure S7. A transimpedance amplifier is used to interface the classifier and the OECN artificial neuron. Depending on the reading of the classifier, the amplifier delivers either a positive or negative current to the OECN.

Classifier Wafer Layout

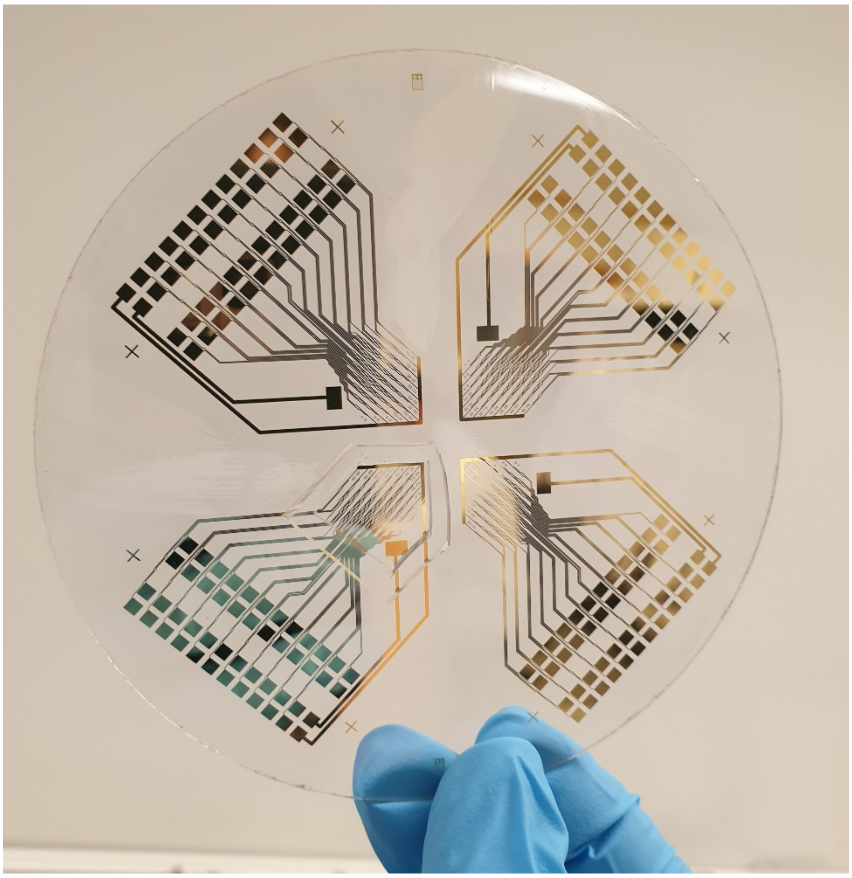

Figure S8. Glass wafer patterned with four EOECT classifiers.

Current drift on positive gate voltages

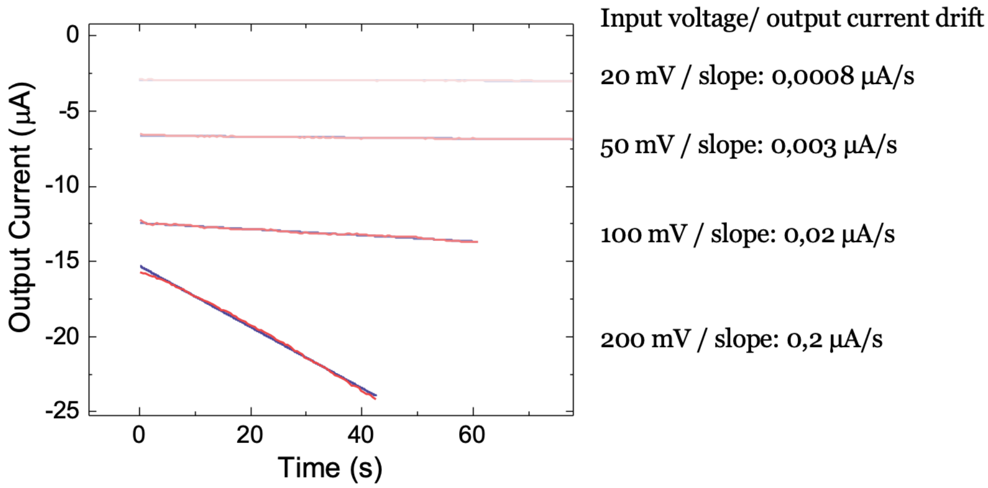

Figure S9. Evaluation of the drift upon the application of a positive read voltage in a solution of 1 mg/mL ETE-S with an applied gate voltage of 0 V.

Pixel Cycling

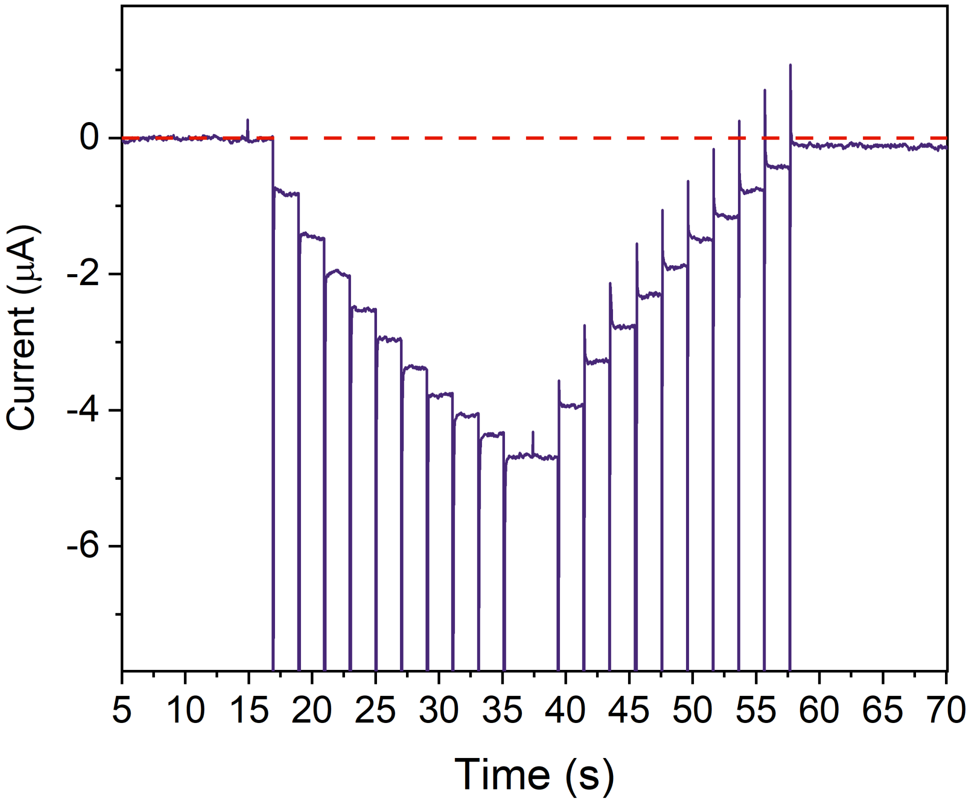

Figure S10. Pixel cycling. A single pixel was evaluated as the weight was bidirectionally modulated by first changing the conductance of the negative coefficient transistor with a series of 10 growth pulses and then changing the conductance of the positive coefficient transistor with a series of 10 growth pulses. Each growth pulse of 0.5V was applied for 100 ms. A linear baseline was subtracted from the data to account for baseline drift due to evaporation.

Output Curves

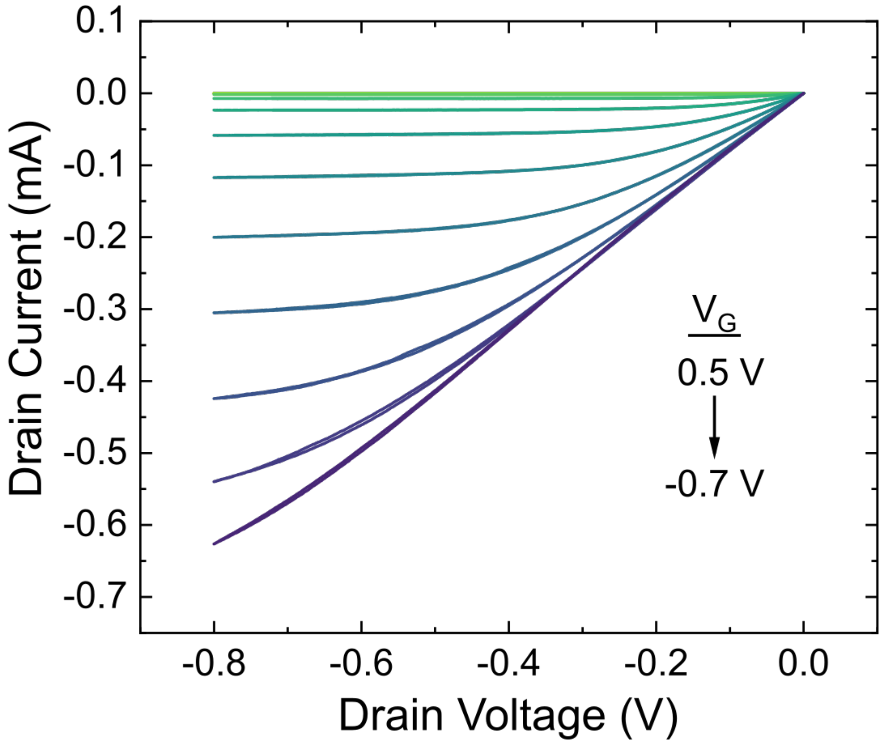

Figure S11. Output characterization of the device in Figure 2b, acquired in a solution of 100 mM NaCl.

[1] B. Widrow, M. E. Hoff, in 7960 IRE WESCON Conv. Record, Stanford Univ Ca Stanford Electronics Labs, 1960, 96.
